# Species diversity of *Derxomyces* (Bulleribasidiaceae, Tremellales) in China, with descriptions of two new species

**DOI:** 10.3897/mycokeys.127.178322

**Published:** 2026-01-22

**Authors:** Zhi-Wen Xi, Rui-Xiu Wang, Chun-Yue Chai, Feng-Li Hui

**Affiliations:** 1 School of Life Science, Nanyang Normal University, Nanyang 473061, China Research Center of Henan Provincial Agricultural Biomass Resource Engineering and Technology, Nanyang Normal University Nanyang China; 2 Research Center of Henan Provincial Agricultural Biomass Resource Engineering and Technology, Nanyang Normal University, Nanyang 473061, China School of Life Science, Nanyang Normal University Nanyang China

**Keywords:** Basidiomycetes, phylogenetic analysis, plant leaves, taxonomy

## Abstract

*Derxomyces* is a monophyletic genus within the family Bulleribasidiaceae, with species that are both abundant and diverse in China, particularly in tropical and subtropical regions. In this study, molecular phylogenetic data and phenotypic characteristics were integrated to investigate the species diversity of *Derxomyces* in Hainan and Henan Provinces of China. A total of nine species were documented, including two new species, *D. alseodaphnes***sp. nov**. (holotype GDMCC 2.534^T^) and *D. henanensis***sp. nov**. (holotype GDMCC 2.336^T^), as well as one species new to China, *D. schimicola*. The remaining species included five that were originally described from China and one that was first identified from New Zealand. Detailed descriptions and illustrations of the two new species are presented, along with comparisons to closely-related species. This study contributes to our understanding of the *Derxomyces* species diversity in China and lays the foundation for future taxonomic and ecological research.

## Introduction

The genus *Derxomyces* (Bulleribasidiaceae, Tremellales) was established by Wang and Bai (2008), with *D. mrakii* (Hamamoto & Nakase) F.-Y. Bai & Q.-M. Wang as its type species. Following its classification, *Derxomyces* has been extensively studied, with a substantial increase in the number of recognised species, based on phylogenetic analyses and phenotypic characteristics (Liu et al. 2012; Li et al. 2020; Jiang et al. 2024). As of now, 39 *Derxomyces* taxa are recognised, including 12 anamorphic species previously known as *Bullera* spp. (Hamamoto and Nakase 1996; Sugita et al. 1999; Bai et al. 2001, 2003; Wang et al. 2004).

The genus is morphologically characterised by cream or yellowish colonies, polar budding and a basal phylogenetic placement within Bulleribasidiaceae. Most *Derxomyces* species produce ballistoconidia, though some may also form hyphae or pseudohyphae (Wang and Bai 2008). *Derxomyces* species lack fermentative ability, possess Q-10 as a predominant ubiquinone and assimilate various carbon sources, but do not utilie hexadecane (Wang and Bai 2008; Li et al. 2020; Jiang et al. 2024).

Species of *Derxomyces* are widely distributed across the globe. The majority of these taxa were initially described from East Asia, with a total of 36 species (Wang and Bai 2008; Liu et al. 2012; Li et al. 2020; Jiang et al. 2024). Additionally, two taxa were first reported from Oceania (Hamamoto and Nakase 1996) and one species was described from North America (Bai et al. 2001). In contrast, research in other regions of the world has been limited. In recent years, 22 *Derxomyces* species have been documented in China, of which 16 were originally described from China, four from Japan, one from Canada and one from New Zealand (Li et al. 2020; Jiang et al. 2024). Despite these contributions, the full extent of *Derxomyces* species diversity in China remains to be explored. During our recent investigations across various locations in China, several *Derxomyces* strains were isolated. In this study, we employed molecular phylogenetic data and phenotypic characteristics to assess the species diversity of *Derxomyces* in China, identifying two previously undescribed species. These species are described and illustrated in detail below.

## Materials and methods

### Sample collection and yeast isolation

Senescent leaf samples were collected from Wuzhi Mountain in Hainan (18°19'N, 109°9'E) and Baotianman Nature Reserve (32°45'N, 113°30'E) in Hean, respectively. Yeast strains were isolated from the leaf surfaces using the ballistoconidia-fall method described by Toome et al. (2013). The senescent leaves were cut into small pieces (30–50 × 40–50 mm) and affixed to the inner lid of a Petri dish with a thin layer of petroleum jelly. The Petri dish contained yeast extract-malt extract (YM) agar medium (0.3% yeast extract, 0.3% malt extract, 0.5% peptone, 1% glucose and 2% agar), supplemented with 0.01% chloramphenicol to prevent bacterial growth. The plates were incubated at 20 °C and monitored daily for colony formation. Emerging yeast colonies were transferred on to fresh YM agar plates for further purification. Purified strains were suspended in 20% (v/v) glycerol and stored at −80 °C for long-term preservation.

### Phenotypic characterisation

Morphological, physiological and biochemical characteristics were examined following standardised methods established by Kurtzman et al. (2011). Colony morphology was observed on YM agar after 7 days of incubation at 20 °C. Cell morphology was examined in YM broth after 3 days of incubation at 20 °C using a LEICA DM2500 microscope (LEICA, Wetzlar, Germany) with LAS V4.13 software. The ballistoconidium-forming activity of all new species was assessed using the inverted-plate method (do Carmo-Sousa and Phaff 1962) on corn meal agar (CMA; 2.5% corn meal infusion and 2% agar) at 20 °C. After 3 to 14 days, discharged spores were collected on a glass slide and examined microscopically. Potential sexual reproduction was investigated for individual strains and strain pairs on CMA, potato dextrose agar (PDA; 20% potato infusion, 2% glucose and 2% agar) and V8 agar (10% V8 juice and 2% agar) at 17 °C for up to two months, with observations made at two-week intervals (Li et al. 2020; Jiang et al. 2024). Glucose fermentation was tested in liquid medium using Durham fermentation tubes. Carbon and nitrogen assimilations were assessed in liquid media, with nitrogen assimilation tests performed using starved inoculum (Kurtzman et al. 2011). Growth at different temperatures (15, 20, 25, 30, 35 and 37 °C) was evaluated on YM agar plates. All new taxonomic descriptions and proposed names were deposited in the MycoBank database (http://www.mycobank.org; 10 November 2025).

### DNA extraction, PCR amplification and sequencing

Genomic DNA was extracted from actively growing yeast cells cultured on YM agar using the Ezup Column Yeast Genomic DNA Purification Kit, following the manufacturer’s protocol (Sangon Biotech Co., Shanghai, China). Two nuclear loci were sequenced: the ITS region and the D1/D2 domain of the LSU rRNA gene, using primer pairs ITS1/ITS4 (White et al. 1990) and NL1/NL4 (Kurtzman and Robnett 1998), respectively. PCR amplification was carried out in a 25 µl reaction volume consisting of 1 µl of DNA template (20 ng/µl), 1 µl of each primer (10 µM), 12.5 µl of Taq 2X PCR Master Mix with blue dye (containing 0.05 u/µl Taq DNA polymerase, 4 mM MgCl_2_, 0.4 mM of each dNTP and reaction buffer; Sangon Biotech Co., Shanghai, China) and 9.5 µl of ddH_2_O. The PCR protocol included: an initial denaturation at 98 °C for 2 min, followed by 35 cycles of denaturation at 98 °C for 10 s, annealing at 55 °C for 10 s, elongation at 72 °C for 15 s and a final elongation at 72 °C for 5 min. PCR products were analysed by electrophoresis in 1% agarose gels. Sanger sequencing was performed by Sangon Biotech (Shanghai) Co., Ltd. (Shanghai, China). The identity and accuracy of each sequence were confirmed by comparison with sequences in the GenBank database. Sequence assembly was conducted using BioEdit v.7.1.3.0 (Hall 1999). All newly-generated sequences were deposited in the GenBank database (https://www.ncbi.nlm.nih.gov/genbank/).

### Phylogenetic analyses

The sequences generated in this study, along with those obtained from previous work and deposited in the GenBank database, were used in our phylogenetic analyses (Table 1). Sequences of the two gene fragments (ITS and LSU) were aligned separately using MAFFT v.7.110 (Katoh and Standley 2013), followed by manual adjustments to remove ambiguous regions using BioEdit v.7.1.3.0 (Hall 1999). The aligned datasets were concatenated using PhyloSuite v.1.2.3 (Zhang et al. 2020).

**Table 1.** GenBank accession numbers and details of isolates chosen for the phylogenetic studies. The newly-generated sequences are indicated in bold and ex-type strains are indicated with ^T^ after the strain number.

Species	Strain no.	Locality	GenBank accession no.	
			ITS	LSU D1/D2
***Derxomyces alseodaphnes***	**NYNU 24817^T^**	**China**	**PQ568969**	**PQ568968**
***Derxomyces alseodaphnes***	**NYNU 2487**	**China**	**PV404185**	**PV404184**
*Derxomyces amylogenes*	CBS 12233^T^	China	NR_157460	NG_059148
*Derxomyces anomalus*	CBS 9607^T^	China	KY103324	NG_059093
*Derxomyces bambusicola*	CGMCC 2.4411^T^	China	NR_158379	HQ890376
*Derxomyces bifurcus*	CGMCC 2.3470^T^	China	MK050319	MK050319
*Derxomyces boekhoutii*	CGMCC 2.3758^T^	China	NR_137702	NG_059099
*Derxomyces boninensis*	JCM 10570^T^	Japan	NR_111013	AY487568
*Derxomyces corylopsis*	CGMCC 2.4409^T^	China	NR_158378	HQ890374
*Derxomyces cylindricus*	CBS 9744^T^	China	NR_121300	NG_059149
*Derxomyces elongatus*	CGMCC 2.3561^T^	China	NR_174747	MK050311
*Derxomyces foliicola*	CGMCC 2.6872^T^	China	OP470246	OP470150
*Derxomyces hainanensis*	CGMCC 2.3467^T^	China	NR_137007	NG_059100
***Derxomyces henanensis***	**NYNU 2211270^T^**	**China**	**OP954741**	**OP954742**
***Derxomyces henanensis***	**NYNU 2210175**	**China**	**OP954655**	**OP954654**
***Derxomyces henanensis***	**NYNU 2211264**	**China**	**PV404187**	**PV404186**
*Derxomyces huiaensis*	JCM 8933^T^	New Zealand	NR_111011	NG_059059
*Derxomyces hubeiensis*	CBS 9747^T^	China	NR_111159	NG_042403
*Derxomyces komagatae*	CBS 10153^T^	Japan	KY103328	NG_059095
*Derxomyces longicylindricus*	CGMCC 2.5660^T^	China	NR_174740	MK050300
*Derxomyces longiovatus*	CGMCC 2.3535^T^	China	NR_174748	MK050312
*Derxomyces linzhiensis*	CGMCC 2.2668^T^	China	NR_137703	NG_059094
*Derxomyces melastomatis*	CGMCC 2.3459^T^	China	NR_174743	MK050305
***Derxomyces melastomatis***	**NYNU 22112**	China	**PV400530**	**PV400529**
*Derxomyces motuoensis*	CGMCC 2.6874^T^	China	OP470247	OP470151
*Derxomyces mrakii*	JCM 8934^T^	New Zealand	NR_111012	KY107621
***Derxomyces mrakii***	**NYNU 2210157**	China	**PV400654**	**PV400652**
*Derxomyces nakasei*	CBS 9746^T^	China	NR_111158	NG_066179
***Derxomyces nakasei***	**NYNU 221087**	China	**PV400544**	**PV400543**
*Derxomyces napiformis*	CGMCC 2.4446^T^	China	NR_174750	MK050321
*Derxomyces orientalis*	CGMCC 2.6871^T^	China	OP470244	OP470148
*Derxomyces ovatus*	CGMCC 2.3572^T^	China	NR_174741	MK050302
*Derxomyces paracylindricus*	CGMCC 2.6875^T^	China	OP470248	OP470152
*Derxomyces polymorphus*	CGMCC 2.4437^T^	China	NR_174745	MK050309
*Derxomyces pseudohuiaensis*	JCM 5984^T^	Japan	NR_111059	NG_059150
*Derxomyces pseudoboekhoutii*	CGMCC 2.4436^T^	China	NR_174746	MK050310
*Derxomyces pseudoschimicola*	CBS 7354^T^	Canada	KY103333	NG_059151
*Derxomyces pseudoyunnanensis*	CGMCC 2.3563^T^	China	NR_174749	MK050313
*Derxomyces pseudocylindricus*	CBS 10826^T^	China	NR_158374	NG_059101
*Derxomyces qinlingensis*	CGMCC 2.2446^T^	China	NR_137008	NG_059096
*Derxomyces schimicola*	CBS 9144^T^	China	NR_167939	KY107626
***Derxomyces schimicola***	**NYNU 224102**	**China**	**PV400559**	**PV400560**
*Derxomyces simaoensis*	CGMCC 2.3571^T^	China	NR_137704	NG_059097
*Derxomyces taiwanicus*	CGMCC 2.4429^T^	China	NR_174742	MK050303
***Derxomyces taiwanicus***	**NYNU 24859**	**China**	**PV400538**	**PV400535**
*Derxomyces waltii*	JCM 10575^T^	Japan	NR_111014	NG_059102
*Derxomyces wuzhishanensis*	CGMCC 2.3760^T^	China	NR_137009	NG_066158
***Derxomyces wuzhishanensis***	**NYNU 24854**	**China**	**PV400534**	**PV400532**
*Derxomyces xingshanicus*	CGMCC 2.2459^T^	China	NR_174744	MK050308
*Derxomyces yunnanensis*	CGMCC 2.3562^T^	China	NR_137010	NG_059098
***Derxomyces yunnanensis***	**NYNU 24881**	**China**	**PV400541**	**PV400539**
*Derxomyces* sp.	2 XZL-2021-1412	China	OL898902	OL898902
*Derxomyces* sp.	2 XZL-2021-0372	China	OL898901	OL898901
*Derxomyces* sp.	1 XZL-2021-0872	China	OL898900	OL898900
*Derxomyces* sp.	1 XZL-2021-0871	China	OL898899	OL898899
*Dioszegia aurantiaca*	CBS 6980^T^	China	NR_155060	NG_059153
*Dioszegia crocea*	CBS 6714^T^	UK	NR_155062	AF075508
*Hannaella coprosmae*	CBS 8284^T^	New Zealand	NR_165939	NG_057682
*Hannaella oryzae*	CBS 7194^T^	Japan	NR_165938	AF075511
*Hannaella surugaensis*	JCM 11903^T^	Japan	NR_163502	NG_058303
*Hannaella urticae*	CGMCC 2.6905^T^	China	OP470256	OP470160

Phylogenetic analysis, based on single LSU or ITS sequences, was performed using evolutionary distance data calculated from Kimura’s two-parameter model with the Neighbour-Joining (NJ) algorithm in MEGA v.7 (Kimura 1980; Kumar et al. 2016; Lachance 2022). Bootstrap analysis was conducted with 1,000 random re-samplings (Felsenstein 1985). Maximum Likelihood (ML) and Bayesian Inference (BI) analyses were performed on the concatenated LSU and ITS sequences using RAxML v.8.2.3 with 1,000 rapid bootstrap replicates (Stamatakis 2014) and MrBayes v.3.2.7a with 5,000,000 generations (Ronquist et al. 2012), respectively. The optimal nucleotide substitution model was determined using MrModelTest v.2.3 (Posada and Crandall 1998), with the GTR + I + G model selected for both the ML and BI analyses. Branches with bootstrap support (BS) values ≥ 50 and Bayesian posterior probabilities (BPPs) ≥ 0.95 were considered significantly supported in all phylogenetic trees constructed in this study.

## Results

### Phylogeny

In this study, 56 yeast strains were isolated from 22 leaf samples collected in Hainan and Henan Provinces, China. Based on ITS and LSU sequence analyses, the isolates were assigned to 14 previously described species — *Bulleribasidium foliicola*, *Cystofilobasidium macerans*, *Derxomyces melastomatis*, *Derxomyces mrakii*, *Derxomyces nakasei*, *Derxomyces schimicola*, *Derxomyces taiwanicus*, *Derxomyces wuzhishanensis*, *Derxomyces yunnanensis*, *Hannaella phyllophila*, *Sporobolomyces bannaensis*, *Sporobolomyces roseus*, *Symmetrospora marina*, *Symmetrospora symmetrica* — and two *Derxomyces* taxa that have not yet been formally described and, therefore, likely represent novel species.

Twelve strains preliminarily identified as *Derxomyces* were subjected to further analyses. The concatenated ITS and LSU dataset consisted of 122 sequences, including 61 ITS sequences and 61 LSU sequences, with 24 sequences newly generated in this study. These sequences represent 61 strains from 49 taxa, with *Dioszegia aurantiaca* L.B. Connell, Redman, R.J. Rodr. & Á. Fonseca and *Dioszegia crocea* (Buhagiar) M. Takash., T. Deák & Nakase serving as the outgroup species.

**Figure 1.**
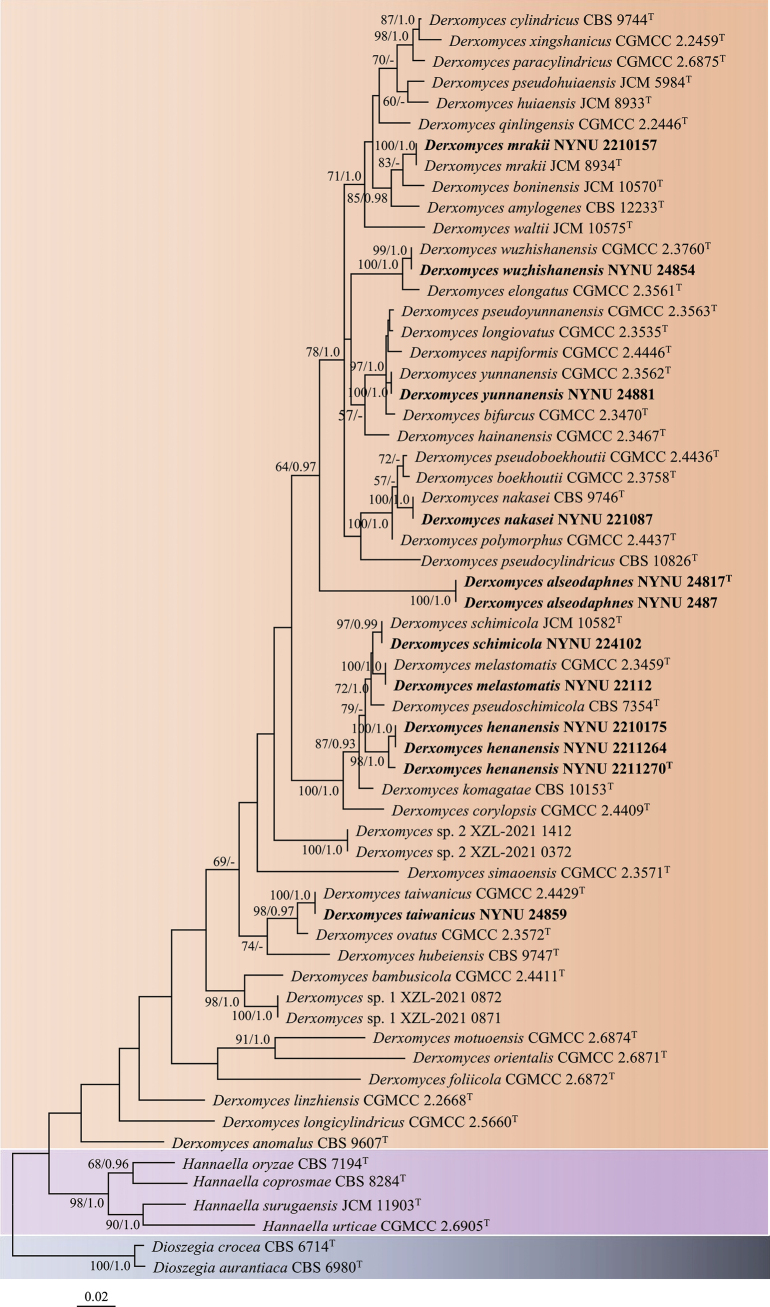
Maximum Likelihood (ML) phylogram of *Derxomyces* inferred from the combined ITS and LSU sequences. The tree is rooted with *Dioszegia aurantiaca* and *Dioszegia crocea* as the outgroup. Bootstrap values ≥ 50 and Bayesian posterior probabilities ≥ 0.95 are indicated on the branches. Sequences generated in this study are shown in bold and ex-type strains are indicated with ^T^ after the strain number.

The phylogenetic trees inferred from both ML and BI analyses showed identical topologies. Therefore, only the tree generated from the ML analysis is presented, with BS ≥ 50% and BPPs ≥ 0.95 indicated on the branches (Fig. 1). The 12 isolates from China were clustered into nine distinct lineages within the *Derxomyces* clade, consistent with the single LSU or ITS dataset phylogeny (Suppl. materials 1, 2). Seven of these lineages corresponded to previously described species: *D. melastomatis* Q.M. Wang, F.Y. Bai & A.H. Li, *D. mrakii* (Hamamoto & Nakase) F.Y. Bai & Q.M. Wang, *D. nakasei* F.Y. Bai, Q.M. Wang & M. Takash. ex F.Y. Bai & Q.M., *D. schimicola* (Sugita, Gibas, M. Takash. & Nakase) F.Y. Bai & Q.M. Wang, *D. taiwanicus* Q.M. Wang, F.Y. Bai & A.H. Li, *D. wuzhishanensis* F.Y. Bai & Q.M. Wang and *D. yunnanensis* F.Y. Bai & Q.M. Wang. The remaining two lineages were genetically distinct from all known *Derxomyces* species, representing two new species within the *Derxomyces* clade.

Two strains, NYNU 24817 and NYNU 2487, were isolated from the leaves of *Alseodaphne rugosa* and *Polyalthia suberosa*, respectively. Despite being isolated from different plant species, these strains shared identical D1/D2 and ITS sequences. These two strains clustered with *D. hubeiensis* with low support in the tree obtained from the ITS dataset (Suppl. material 2), but they formed a distinct branch in the trees of the LSU and combined ITS and LSU datasets (Fig. 1, Suppl. material 1). They differed from *D. hubeiensis* and other *Derxomyces* species by more than 13 nucleotide (nt) substitutions (2.2%) in the D1/D2 domain and 51 nt mismatches (8.7%) in the ITS region. These findings indicate that strains NYNU 24817 and NYNU 2487 represent a new *Derxomyces* species, for which the name *Derxomyces alseodaphnes* sp. nov. is proposed.

Three strains, NYNU 2211270, NYNU 2210175 and NYNU 2211264, were isolated from the same host plant, *Corydalis balansae* and exhibited similar sequences with three nucleotide differences in the D1/D2 domain and four in the ITS region, indicating conspecificity. These three strains, along with four other known species — *D. komagatae*, *D. pseudoschimicola*, *D. melastomatis* and *D. schimicola* — clustered together in a highly supported branch (Fig. 1, Suppl. materials 1, 2). They differed from the other species in this cluster by three to nine nt substitutions (0.5–1.5%) in the D1/D2 domain and 35–42 nt mismatches (6.2–7.8%) in the ITS region. The results suggest that these three strains represent a new *Derxomyces* species, for which the name *Derxomyces henanensis* sp. nov. is proposed.

### Taxonomy

#### Derxomyces alseodaphnes

Taxon classificationFungiTremellalesBulleribasidiaceae

C.Y. Chai & F.L. Hui sp. nov.

F905EF9B-9804-58F5-AB70-E5AEB8980C1D

861306

Fig. 2

##### Etymology.

The specific epithet *alseodaphnes* refers to *Alseodaphne*, the plant genus from which the type strain was isolated.

**Figure 2.**
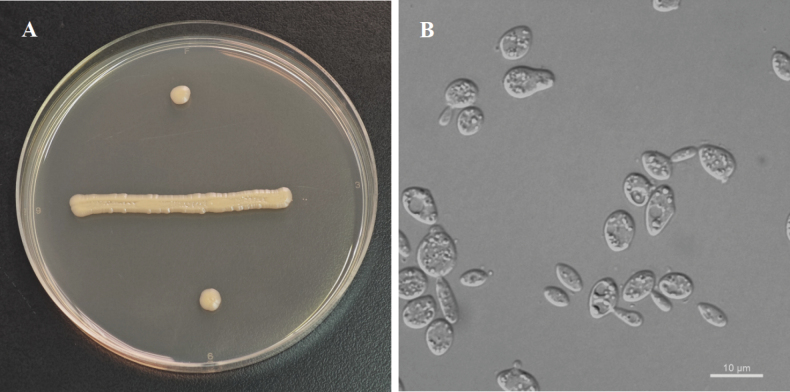
Morphology of *Derxomyces alseodaphnes* (NYNU 24817). **A**. Colony on YM agar after 7 days at 20 °C; **B**. Budding cells in YM broth after 3 days at 20 °C.

##### Typus.

China • Hainan Prov.: Wuzhishan City, Wuzhi Mountain, on the phylloplane of *Alseodaphne rugosa*, August 2024, S.L. Lv, NYNU 24817 (holotype GDMCC 2.534^T^ preserved as a metabolically inactive state, culture ex-type JCM 38177).

##### Description.

On YM agar after 7 days at 20 °C, the streak culture is pale-yellow, butyrous and smooth, with an entire margin. After 3 days in YM broth at 20 °C, cells are ovoid and ellipsoidal, 2.1–4.0 × 2.6–4.9 μm and single, budding is polar. After 1 month at 20 °C, a ring and sediment are present. In Dalmau plate culture on CMA, pseudohyphae and hyphae are not formed. Sexual structures are not observed on PDA, CMA or V8 agar. Ballistoconidia are not produced. Glucose fermentation is absent. Glucose, inulin, sucrose, raffinose, melibiose, galactose, trehalose, maltose, melezitose, methyl-α-D-glucoside, cellobiose, salicin (weak), L-sorbose (delayed), L-rhamnose, D-xylose, L-arabinose, D-arabinose, 5-keto-D-gluconate, D-ribose (delayed), erythritol (delayed), galactitol (delayed), D-mannitol, D-glucitol (delayed), myo-inositol (weak), DL-lactate, succinate (weak), citrate (weak and delayed), D-gluconate, D-glucosamine, N-acetyl-D-glucosamine, 2-keto-D-gluconate, D-glucuronate and glucono-1,5-lactone (weak) are assimilated as sole carbon sources. Lactose, methanol, ethanol, glycerol and ribitol are not assimilated. Ethylamine and L-lysine are assimilated as sole nitrogen sources. Nitrate, nitrite and cadaverine are not assimilated. Growth is observed on YM agar at 25 °C, but not at 30 °C. Growth on 50% (w/w) glucose-yeast extract agar is negative. Growth in vitamin-free medium is positive. Starch-like substances are not produced. Urease activity is positive. Diazonium Blue B reaction is positive.

##### Additional strain examined.

China • Hainan Prov.: Wuzhishan City, Wuzhi Mountain, on the phylloplane of *Polyalthia suberosa*, August 2024, S.L. Lv, NYNU 2487.

##### GenBank accession numbers.

Holotype GDMCC 2.534^T^ (ITS: PQ568969, D1/D2: PQ568968); additional strain NYNU 2487 (ITS: PV404185, D1/D2: PV404184).

##### Note.

Physiologically, *D. alseodaphnes* can be distinguished from the closely-related species, *D. hubeiensis*, in its inability to assimilate ribitol and grow at 30 °C, while being able to assimilate inulin and to grow in vitamin-free medium (Table 2).

**Table 2.** Physiological and biochemical characteristics that differ between the new species and closely-related species.

Characteristics	1	2*	3	4*	5*	6*
Assimilation						
Inulin	+	–	–	–	+	–
Trehalose	+	+	–	+	+	+
Cellobiose	+	s	–	+	+	s
L-Sorbose	d	s, w	–	s	–	–
Ribitol	–	s	+	s	–	–
D-Mannitol	+	s	–	+	w	s
Myo-Inositol	w	+	–	+	+	s
Growth tests						
Growth in vitamin-free medium	w	–	+	–	–	–
Growth at 30 °C	–	w	–	–	–	–

**Notes**. 1, *D. alseodaphnes*; 2, *D. hubeiensis*; 3, *D. henanensis*; 4, *D. pseudoschimicola*; 5, *D. melastomatis*; 6, *D. komagatae*. +, positive reaction; –, negative reaction; d, delayed positive; s, slow positive; w, weakly positive. All data from this study, except* which were obtained from the original description (Wang and Bai 2008; Jiang et al. 2024).

#### Derxomyces henanensis

Taxon classificationFungiTremellalesBulleribasidiaceae

C.Y. Chai & F.L. Hui sp. nov.

C2EFEFFE-E0BA-5097-914C-D8FB6900E03D

861307

Fig. 3

##### Etymology.

The specific epithet *henanensis* refers to the geographic origin of the type strain, Baotianman Nature Reserve, Nanyang City, Henan Province.

**Figure 3.**
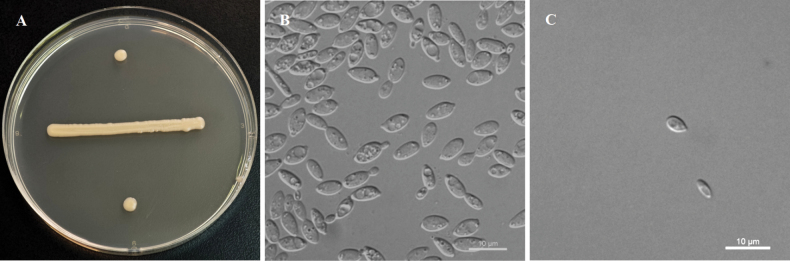
Morphology of *Derxomyces henanensis* (NYNU 2211270). **A**. Colony on YM agar after 3 days at 25 °C; **B**. Budding cells in YM broth after 3 days at 25 °C; **C**. Ballistoconidia on corn meal agar after 15 days at 20 °C.

##### Typus.

China • Henan Prov.: Nanyang City, Baotianman Nature Reserve, on the phylloplane of *Corydalis balansae*, August 2022, J.Z. Li, NYNU 2211270 (holotype GDMCC 2.336^T^ preserved as a metabolically inactive state, culture ex-type PYCC 9941).

##### Description.

On YM agar after 7 days at 20 °C, the streak culture is pale-yellow, butyrous and smooth, with an entire margin. After 3 days in YM broth at 20 °C, cells are ovoid and ellipsoidal, 3.2–3.8 × 6.3–8.5 μm and single, budding is polar. After 1 month at 20 °C, a ring and sediment are present. In Dalmau plate culture on CMA, pseudohyphae and hyphae are not formed. Ballistoconidia are produced on corn meal agar and are ovoid, 2.2–3.5 × 3.5–5.5 μm. Sexual structures are not observed on PDA, CMA or V8 agar. Glucose fermentation is absent. Glucose, sucrose (weak and delayed), raffinose (delayed), melibiose (weak and delayed), galactose (delayed), maltose (weak and delayed), melezitose (weak and delayed), methyl-α-D-glucoside (delayed), salicin (delayed), L-rhamnose (delayed), D-xylose, L-arabinose, D-arabinose, 5-keto-D-gluconate, D-ribose, ribitol, galactitol, D-glucitol (delayed), DL-lactate (delayed), succinate, citrate (weak and delayed), D-glucosamine (weak), N-acetyl-D-glucosamine (delayed), D-glucuronate (weak and delayed) and glucono-1,5-lactone (weak) are assimilated as sole carbon sources. Inulin, lactose, trehalose, cellobiose, L-sorbose, methanol, ethanol, glycerol, erythritol, D-mannitol, myo-inositol, D-gluconate and 2-keto-D-gluconate are not assimilated. L-Lysine is assimilated as the sole nitrogen source. Nitrate, nitrite, ethylamine and cadaverine are not assimilated. Growth is observed on YM agar at 25 °C, but not at 30 °C. Growth on 50% (w/w) glucose-yeast extract agar is negative. Growth in vitamin-free medium is positive. Starch-like substances are not produced. Urease activity is positive. Diazonium Blue B reaction is positive.

##### Additional strain examined.

China • Nanyang City, Baotianman Nature Reserve, on the phylloplane of *Corydalis balansae*, August 202, J.Z. Li, NYNU 2211270 and NYNU 2211264.

##### GenBank accession numbers.

Holotype GDMCC 2.336^T^ (ITS: OP954741, D1/D2: OP954742); additional strains NYNU 2210175 (ITS: OP954655, D1/D2: OP954654) and NYNU 2211264 (ITS: PV404187, D1/D2: PV404186).

##### Note.

Physiologically, *D. henanensis* can be distinguished from the closely-related species *D. pseudoschimicola*, *D. melastomatis* and *D. komagatae* in its inability to assimilate trehalose, cellobiose, D-mannitol and myo-Inositol, while being able to grow in vitamin-free medium (Table 2).

## Discussion

The genus *Derxomyces* exhibits considerable taxonomic richness in China, making it one of the most well-studied genera within the family Bulleribasidiaceae in the country. To date, a total of 40 species have been recorded in China, including the previously reported 37 species and two new species described in this study: *D. alseodaphnes* and *D. henanensis*. Additionally, *D. schimicola*, a species new to China, was also discovered during this research. This study, therefore, adds two new species, six previously known species and one newly-recorded species in China, thus enriching the species diversity of the genus in the country. Moreover, sequence data from public databases suggest the presence of several unpublished strains, including XZL-2021-1412, XZL-2021-0372, XZL-2021-0872 and XZL-2021-0871 from China. These findings imply the potential existence of two additional *Derxomyces* species in China, warranting further investigation.

Species of *Derxomyces* are ballistoconidium-forming yeasts, as inferred from their original classification within *Bullera* (Wang and Bai 2008). Most species in this genus typically produce ballistoconidia, which are identified as an opaque mirror image of the culture formed by the discharged spores on the lid of an inverted Petri dish (do Carmo-Sousa and Phaff 1962; Kurtzman et al. 2011). However, the production of ballistoconidia is influenced by cultivation conditions and varies between clones (Nakase and Takashima 1993; Nakase 2000). In this study, *D. henanensis* sp. nov. produces ovoid ballistoconidia, whereas *D. alseodaphnes* sp. nov. does not. This phenomenon is exceptionally rare within the genus *Derxomyces*, with only *D. pseudoboekhoutii* previously reported as lacking the ability to produce ballistoconidia (Li et al. 2020).

Species of the genus *Derxomyces* are widely distributed in nature and are primarily regarded as epiphytic yeasts associated with plant leaves in tropical and subtropical regions (Wang and Bai 2008; Boekhout et al. 2011; Liu et al. 2012; Li et al. 2020; Jiang et al. 2024). In this study, the five strains representing two novel *Derxomyces* species share the common ecological niche of plant leaves, consistent with most previously described species in the genus. *Derxomyces alseodaphnes* was isolated from two different plant species, *Alseodaphne rugosa* and *Polyalthia suberosa*, collected on Wuzhi Mountain in Hainan Province. *Derxomyces henanensis* was repeatedly recovered from the same host plant, *Corydalis balansae*, collected on Baotianman Mountain in Henan Province. The new record species, *D. schimicola*, was exclusively isolated from *Euonymus alatus* collected on Baotianman Mountain in Henan Province. These findings highlight that *Derxomyces* species are commonly associated with tropical and subtropical plant leaves. Therefore, future studies on *Derxomyces* species diversity should give full consideration to the ecological significance of leaf habitats.

As a result of this study, the genus *Derxomyces* now comprises 41 recognised species. Previous research has shown that certain *Derxomyces* species play a significant role in food and medical fields. These species are known to produce carotenoids and astaxanthin and have been shown to regulate intestinal immune homeostasis in antibiotic-treated mice with diarrhoea (Kong et al. 2021). As a result, interest in these fungi extends beyond taxonomy to their ecological roles and potential applications in agriculture, industry and medicine, underscoring their economic significance.

## Supplementary Material

XML Treatment for Derxomyces alseodaphnes

XML Treatment for Derxomyces henanensis

## Additional information

### Use of AI

No use of AI was reported.

### Author ORCIDs

Zhi-Wen Xi https://orcid.org/0000-0002-5814-5283

Rui-Xiu Wang https://orcid.org/0009-0009-5141-7096

Chun-Yue Chai https://orcid.org/0000-0001-7753-6223

Feng-Li Hui https://orcid.org/0000-0001-7928-3055

### Data availability

All of the data that support the findings of this study are available in the main text or Supplementary Information.

## Supplementary materials

Supplementary material 1 Neighbour-joining (NJ) phylogenetic tree of *Derxomyces* generated from the LSU sequence data Data type pdf https://binary.pensoft.net/file/1517971This dataset is made available under the Open Database License (http://opendatacommons.org/licenses/odbl/1.0/). The Open Database License (ODbL) is a license agreement intended to allow users to freely share, modify, and use this Dataset while maintaining this same freedom for others, provided that the original source and author(s) are credited. Zhi-Wen Xi, Rui-Xiu Wang, Chun-Yue Chai, Feng-Li Hui

Supplementary material 2 Neighbour-joining (NJ) phylogenetic tree of *Derxomyces* generated from the ITS sequence data Data type pdf https://binary.pensoft.net/file/1517972This dataset is made available under the Open Database License (http://opendatacommons.org/licenses/odbl/1.0/). The Open Database License (ODbL) is a license agreement intended to allow users to freely share, modify, and use this Dataset while maintaining this same freedom for others, provided that the original source and author(s) are credited. Zhi-Wen Xi, Rui-Xiu Wang, Chun-Yue Chai, Feng-Li Hui
