# Supplementary figures and images for: Species diversity of Derxomyces (Bulleribasidiaceae, Tremellales) in China, with descriptions of two new species
Source: MycoKeys. 2026 Jan 22;127:155–68. doi: 10.3897/mycokeys.127.178322 (PMC12856483; doi:10.3897/mycokeys.127.178322)

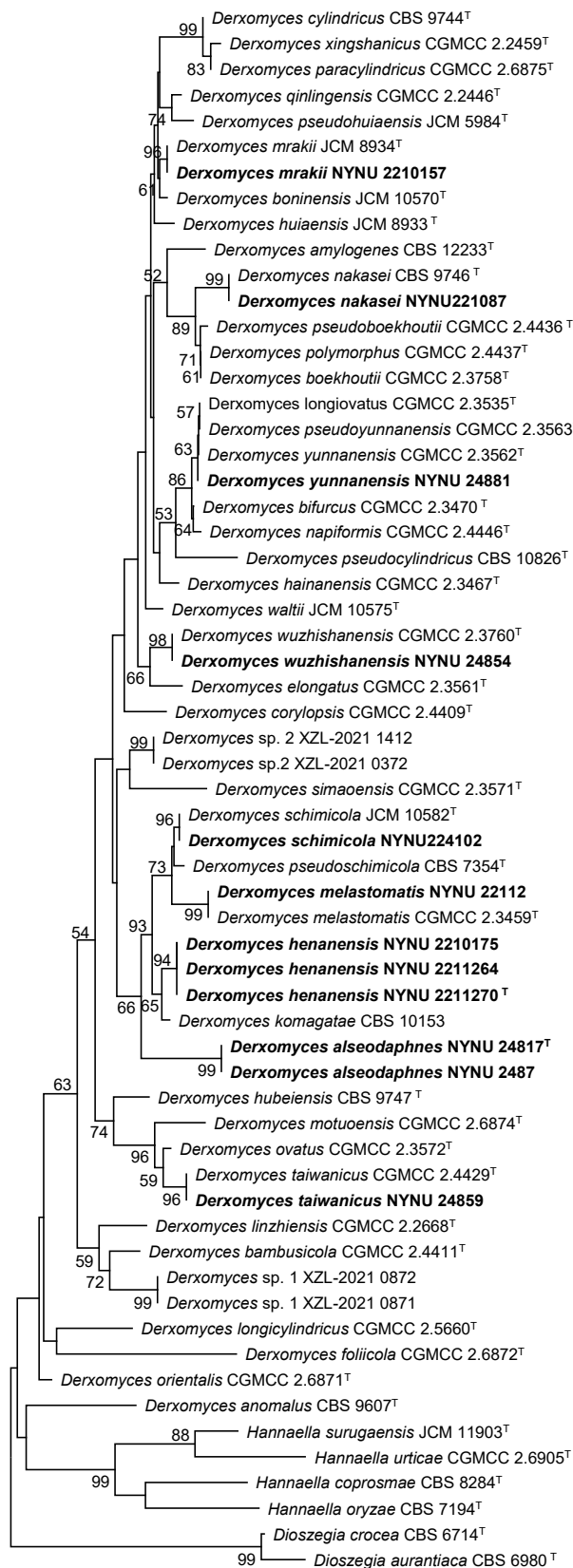

0.02

Supplement: Supplementary material 1 — Neighbour-joining (NJ) phylogenetic tree of Derxomyces generated from the LSU sequence data [file mycokeys-127-155-s001.pdf]

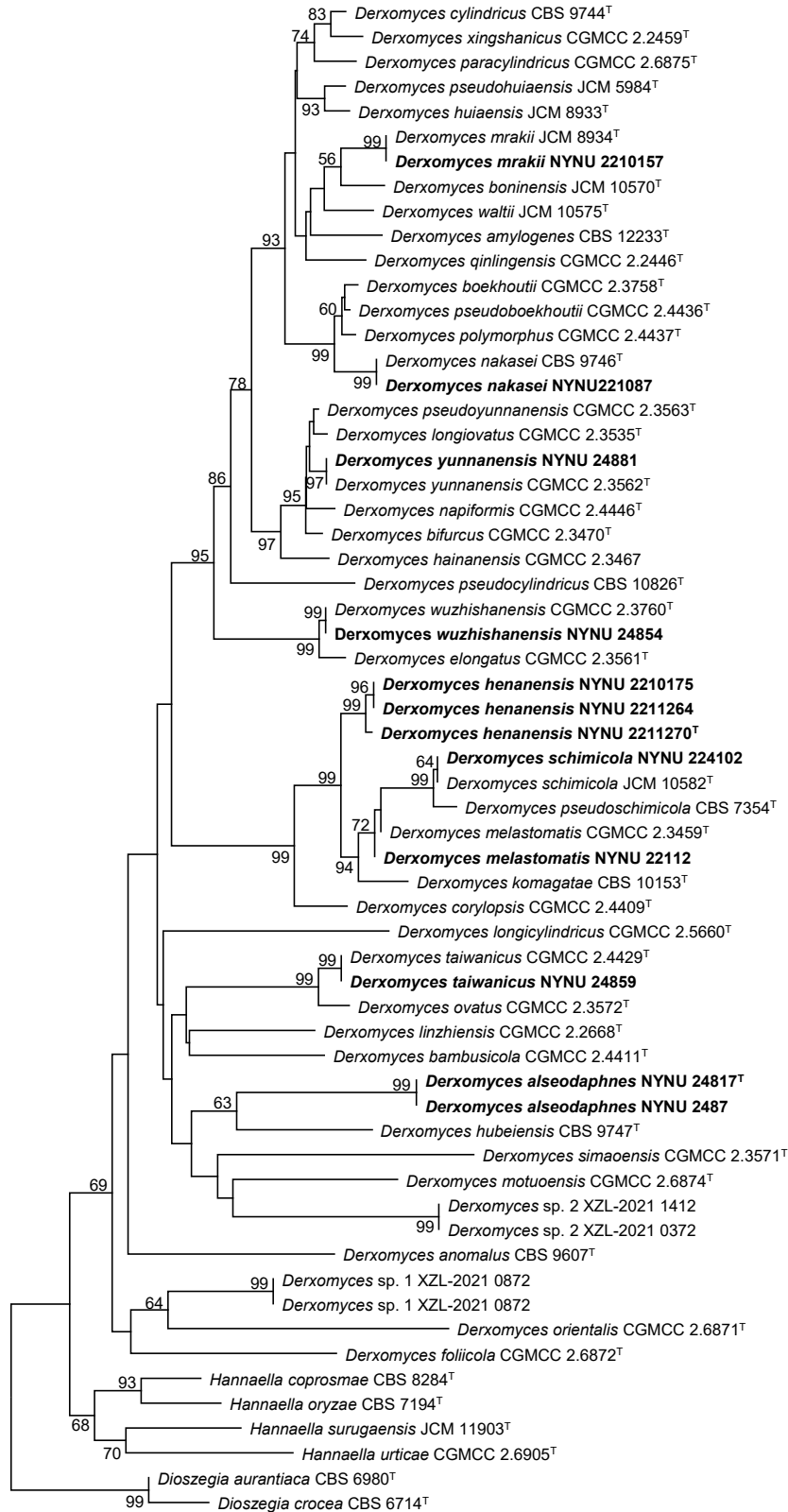

0.02

Supplement: Supplementary material 2 — Neighbour-joining (NJ) phylogenetic tree of Derxomyces generated from the ITS sequence data [file mycokeys-127-155-s002.pdf]
